# Supplementary material for: Occurrence of High Levels of Cefiderocol Resistance in Carbapenem-Resistant Escherichia coli before Its Approval in China: a Report from China CRE-Network
Source: Microbiol Spectr. 2022 Apr 28;10(3):e02670-21. doi: 10.1128/spectrum.02670-21 (PMC9241927; doi:10.1128/spectrum.02670-21)
Supplement: SUPPLEMENTAL FILE 1 — Supplemental material. Download spectrum.02670-21-s001.pdf, PDF file, 0.5 MB [file spectrum.02670-21-s001.pdf]

Figure S1. The phylogenetic tree based on core genome of 20 ST167 cefiderocol resistant *Escherichia coli*

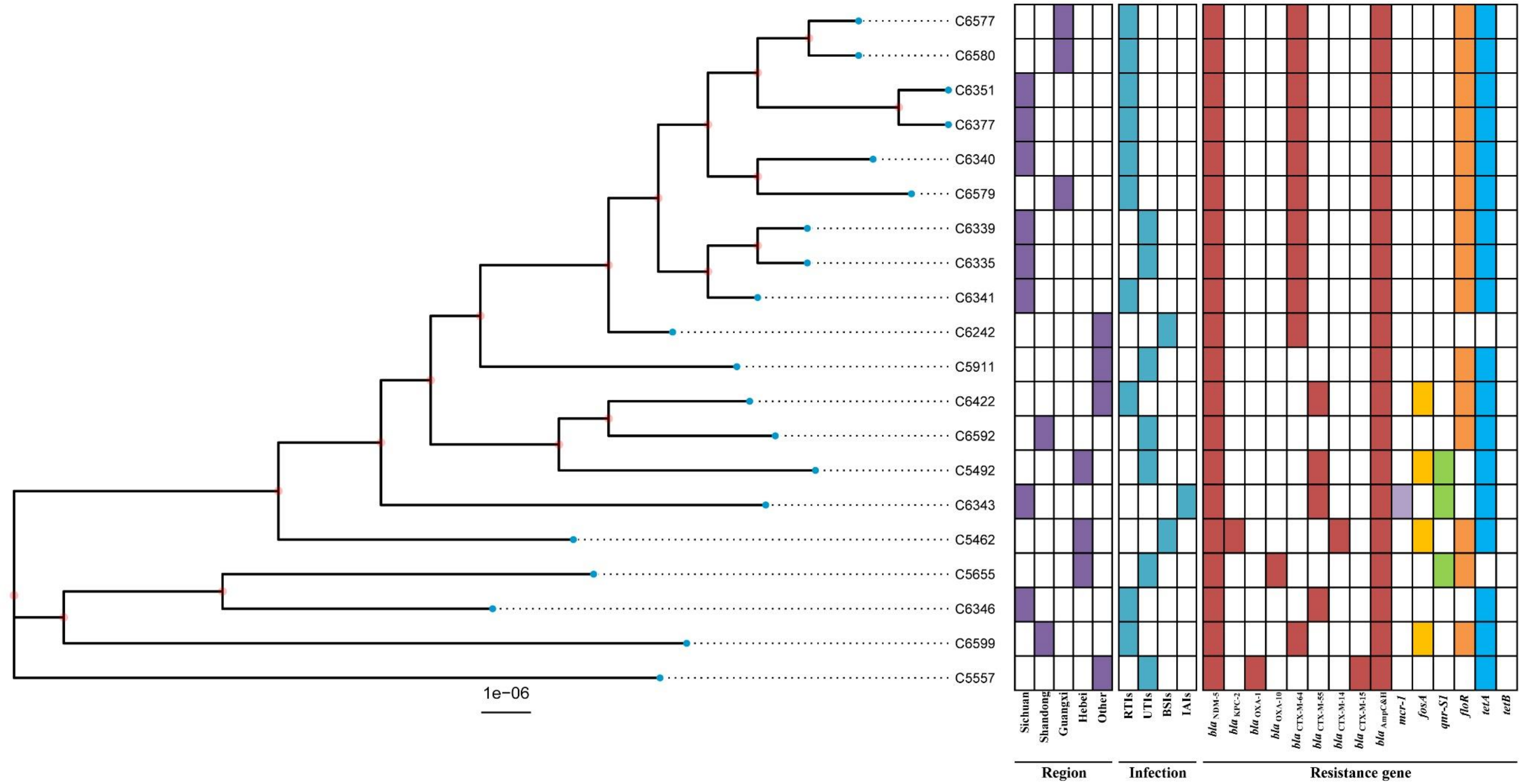

The blank part represents the absence of the corresponding gene, and the other colored parts represent the presence of the corresponding gene.

This figure comprised 4228/6533 core to total genes.

Abbreviations: RTIs: Respiratory tract infection; UTIs: Urinary tract infection; BSIs: Bloodstream infection; IAIs Intra-abdominal infection

Figure S2. Comparison of *bla*$_{NDM}$ copy numbers estimated by second-generation data

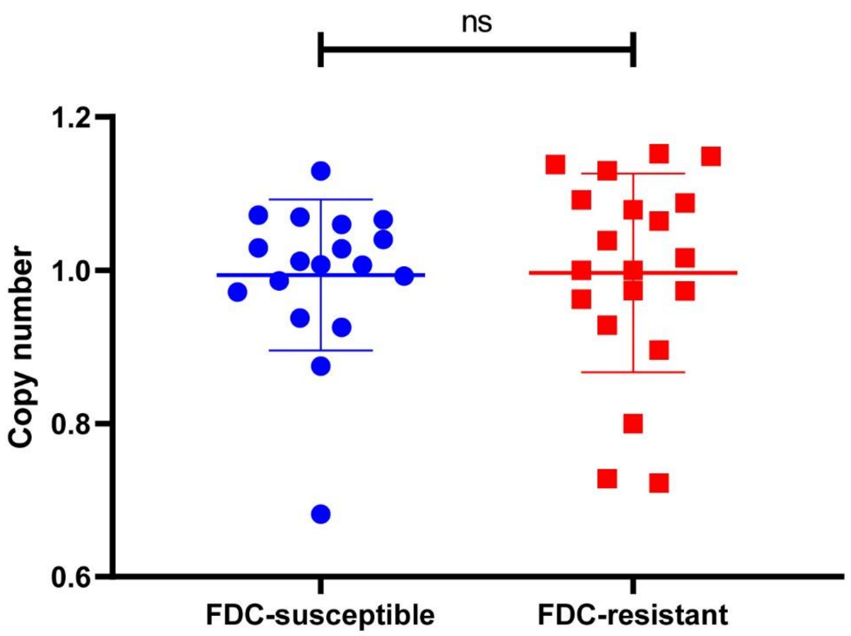

Abbreviations: FDC, cefiderocol

Table S1. List of primers used in this study

Primer	Product	Sequence (5’–3’)	Product length (bp)	Annealing temperature (◦C)
Vector_pEASYT1_cirA_FW	vector	TCGCT TCTGA GCGCG GCGGG TGTGG TGGTT	3595	56
Vector_pEASYT1_cirA_RV		ACCTA AACAT AGCTG TTTCC TGTGT GAAAT		
Insert_pEASYT1_cirA_FW	insert	ACACA GGAAA CAGCT ATGTT TAGGT TGAAC CCTTT	2022	56
Insert_pEASYT1_cirA_RV		CCACA CCCGC CGCGC TCAGA AGCGA TAATC CACTG		
CirA_verification_F	verification primer	GCACTATACCGGGCAGAAAC	685	58
CirA_verification_R		TTCTGCGGACTGGCTTTCTA		
Vector_pEASYT1_pbp3_FW	vector	CAGAT CGTAA GCGCG GCGGG TGTGG TGGTT	3595	56
Vector_pEASYT1_pbp3_RV		CTGCT TTCAT AGCTG TTTCC TGTGT GAAAT		
Insert_pEASYT1_pbp3_FW	insert	ACACA GGAAA CAGCT ATGAA AGCAGCGGCGAAAAC	1809	56
Insert_pEASYT1_pbp3_RV		CCACA CCCGC CGCGC TTACG ATCTG CCACC TGTCC		
PBP3_verification_F	verification primer	ACCCTGACCGGGGTATTACA	931	58
PBP3_verification_R		GCCCACTACGTGAACCATCA		
cirA_spacer_F:	spacer	TAGT GAAACGCGCCGACAGCGCGA	20	
cirA_spacer_R:		AAAC TCGCGCTGTCGGCGCGTTTC		
HR_cirA_F1:	repair arm 1	cgagtcggtgcttttttgatatcg CAACACAACAAGGAGCCACG	889	58
HR_cirA_R1:		GTAGTTACCTCATGGAGATATGG TGAGAAGATGCCCGGCGA		
HR_cirA_F2:	repair arm 2	TCGCCGGGCATCTTCTCA CCATATCTCCATGAGGTAACTAC	812	58
HR_cirA_R2:		actagtggatcccccgggctgcagg TATGCGTCTACTCGTATGCT		
NDM_spacer_F:	spacer	ATTGGCCAGCAAATGGAAAC	20	
NDM_spacer_R:		GTTTCCATTTGCTGGCCAAT		

Table S2. Sequence alignment results of penicillin binding protein of 26 strains cefiderocol-resistant *Escherichia coli*

Isolate No.	MLST	PBP1a	PBP1a	PBP1c	PBP3	PBP2/PBP4/PBP5	PBP6	PBP7
C5462	167	WT	WT	WT	Q227H,333 aa intertion YRIN,E349K,I532L	WT	WT	WT
C5492	167	WT	WT	WT	Q227H,333 aa intertion YRIN,E349K,I532L	WT	WT	WT
C5557	167	WT	WT	WT	Q227H,333 aa intertion YRIN,E349K,I532L	WT	WT	WT
C5655	167	WT	WT	WT	Q227H,333 aa intertion YRIN,E349K,I532L	WT	WT	WT
C5881	405	Q361D,S497G	33Ddel	D60E,A202T,T273V,S336A,E446G,S500G,N578D,T581S,R602L,R669H,S680I	333 aa intertion YRIK,A413V	WT	V383M	WT
C5911	167	WT	WT	WT	Q227H,333 aa intertion YRIN,E349K,I532L	WT	WT	WT

C6242	167	WT	WT		WT	Q227H,333 aa intertion YRIN,E349K,I532L	WT	WT	WT
C6335	167	WT	WT		WT	Q227H,333 aa intertion YRIN,E349K,I532L	WT	WT	WT
C6339	167	WT	WT		WT	Q227H,333 aa intertion YRIN,E349K,I532L	WT	WT	WT
C6340	167	WT	WT		WT	Q227H,333 aa intertion YRIN,E349K,I532L	WT	WT	WT
C6341	167	WT	WT		WT	Q227H,333 aa intertion YRIN,E349K,I532L	WT	WT	WT
C6343	167	WT	WT		WT	Q227H,333 aa intertion YRIN,E349K,I532L	WT	WT	WT
C6346	167	WT	WT		WT	Q227H,333 aa intertion	WT	WT	WT

					YRIN,E349K,I532L			
C6351	167	WT	WT	WT	Q227H,333 aa intertion YRIN,E349K,I532L	WT	WT	WT
C6352	410	Q361D,E377D,S497G	WT	R86H,T273V,S336A,Q495K,S680I,Q689K	Q227H,333 aa intertion YRIN,E349K,I532L	WT	V383M	WT
C6377	167	WT	WT	WT	Q227H,333 aa intertion YRIN,E349K,I532L	WT	WT	WT
C6422	167	WT	WT	WT	Q227H,333 aa intertion YRIN,E349K,I532L	WT	WT	WT
C6575	617	WT	WT	D60E,A202T,T273V,S336A,E446G,S500G,N578D,T581S,R602L,R669H,S680I	Q227H,333 aa intertion YRIN,E349K,I532L	WT	WT	F19L
C6577	167	WT	WT	WT	Q227H,333 aa intertion YRIN,E349K,I532L	WT	WT	WT

C6579	167	WT	WT	WT	Q227H,333 aa intertion YRIN,E349K,I532L	WT	WT	WT
C6580	167	WT	WT	WT	Q227H,333 aa intertion YRIN,E349K,I532L	WT	WT	WT
C6592	167	WT	WT	WT	Q227H,333 aa intertion YRIN,E349K,I532L	WT	WT	WT
C6599	167	WT	WT	WT	Q227H,333 aa intertion YRIN,E349K,I532L	WT	WT	WT
C6617	11738	WT	WT	D60E,A202T,T273V,S336A,E446G,S500G,N578D,T581S,R602L,R669H,S680I	Q227H,333 aa intertion YRIN,E349K,I532L	WT	WT	F19L
C6619	746	Q361D,E377D,S497G	WT	WT	Q227H,333 aa intertion YRIN,E349K,I532L	WT	A104T	F19L

C6620	746	Q361D,E377D,S497G	WT	WT	Q227H,333 aa intertion YRIN,E349K,I532L	WT	A104T	F19L

Table S3. Sequence alignment results of TDBTs of 26 strains cefiderocol-resistant *Escherichia coli*

Isolate No.	MLST	FhuA	FhuE	FecA	FepA	CirA	Fiu	BtuB
C5462	167	P50S	WT	T16A,V217I,R365H,L444V	K69E	truncated at 109 aa	WT	WT
C5492	167	P50S	WT	T16A,V217I,R365H,L444V	K69E	truncated at 109 aa	WT	WT
C5557	167	P50S	WT	T16A,V217I,R365H,L444V	K69E	truncated at 109 aa	WT	WT
C5655	167	P50S	WT	T16A,V217I,R365H,L444V	K69E	truncated at 109 aa	WT	WT

C5881	405	S216A,N434D,D698E,	A26V,I220F,S272N,Q317R,T551I,T717A	T16A,A190T,L444V	K69E,A312S,T420A	truncated at 621 aa	V211A,V235I,T367A,G388A,T493A,V495M,M513V	C11F,V127I,I325T,S330N,T341S,T343A,G347A,E350K,D431N
C5911	167	P50S	WT	T16A,V217I,R365H,L444V,E517K	K69E	truncated at 109 aa	WT	WT
C6242	167	P50S	WT	T16A,V217I,R365H,L444V	K69E	truncated at 109 aa	WT	WT
C6335	167	P50S	WT	T16A,V217I,R365H,L444V	K69E	truncated at 109 aa	WT	WT
C6339	167	P50S	WT	T16A,V217I,R365H,L444V	K69E	truncated at 109 aa	WT	WT
C6340	167	P50S	WT	T16A,V217I,R365H,L444V	K69E	truncated at 109 aa	WT	WT
C6341	167	P50S	WT	T16A,V217I,R365H,L444V	K69E	truncated at 109 aa	WT	WT
C6343	167	P50S	WT	T16A,V217I,R365H,L444V	K69E	truncated at 109 aa	WT	WT

C6346	167	P50S	WT	T16A,V217I,R365H,L444V	K69E	truncated at 109 aa	WT	WT
C6351	167	P50S	WT	T16A,V217I,R365H,L444V	K69E	truncated at 109 aa	WT	WT
C6352	410	P50S	Q317R,S389I,G725S	T16A,N186S	K69E,S293A,A312S,I377L,T420A	WT	WT	D351N
C6377	167	P50S	WT	T16A,V217I,R365H,L444V	K69E	truncated at 109 aa	WT	WT
C6422	167	P50S	WT	T16A,V217I,R365H,L444V	K69E	truncated at 109 aa	WT	WT
C6575	617	WT	WT	T16A,V217I,R365H,L444V	K69E	truncated at 389 aa	WT	WT
C6577	167	P50S	WT	T16A,V217I,R365H,L444V	K69E	truncated at 109 aa	WT	WT
C6579	167	P50S	WT	T16A,V217I,R365H,L444V	L45I,K69E	truncated at 109 aa	WT	WT
C6580	167	P50S	WT	T16A,V217I,R365H,L444V	K69E	truncated at 109 aa	WT	WT

C6592	167	P50S	WT	T16A,V217I,R365H,L444V	K69E	truncated at 109 aa	WT	WT
C6599	167	P50S	WT	T16A,V217I,R365H,L444V	K69E	truncated at 109 aa	WT	WT
C6617	11738	WT	WT	T16A,V217I,R365H,L444V	K69E	WT	WT	WT
C6619	746	V739F	WT	T16A,V217I,R365H,L444V	P25S,K69E,T420A	WT	L254M	truncated at 154 aa
C6620	746	V739F	WT	T16A,V217I,R365H,L444V	P25S,K69E,T420A	WT	L254M	truncated at 154 aa

Table S4. Sequencing statistics

Sample ID	species	Bioproject Number (NCBI)	Accession Number (NCBI)	contig_count	N50	largest_contig	total_size
C5462	Escherichia coli	PRJNA756960	SAMN20930911	679	88273	518563	5167926
C5492	Escherichia coli	PRJNA756960	SAMN20930912	521	93769	456592	5128411
C5557	Escherichia coli	PRJNA756960	SAMN20930913	420	106347	353881	4979093
C5655	Escherichia coli	PRJNA756960	SAMN20930914	774	94530	329102	5415733
C5881	Escherichia coli	PRJNA756960	SAMN20930915	445	80931	379078	5535497
C5911	Escherichia coli	PRJNA756960	SAMN20930916	408	95184	531587	4986317
C6242	Escherichia coli	PRJNA756960	SAMN20930917	517	83663	496464	5104817
C6335	Escherichia coli	PRJNA756960	SAMN20930918	610	95184	496464	5207731
C6339	Escherichia coli	PRJNA756960	SAMN20930919	473	88732	496464	5180014
C6340	Escherichia coli	PRJNA756960	SAMN20930920	469	82753	392314	5126949
C6341	Escherichia coli	PRJNA756960	SAMN20930921	434	93770	496466	5175124
C6343	Escherichia coli	PRJNA756960	SAMN20930922	295	84357	367033	5164327
C6351	Escherichia coli	PRJNA756960	SAMN20930924	430	93769	496463	5087528
C6352	Escherichia coli	PRJNA756960	SAMN20930925	1664	115352	305105	5280152
C6377	Escherichia coli	PRJNA756960	SAMN20930926	460	93770	496463	5097396
C6422	Escherichia coli	PRJNA756960	SAMN20930927	424	93770	470189	5013720
C6575	Escherichia coli	PRJNA756960	SAMN20930928	747	83595	229950	5200606
C6579	Escherichia coli	PRJNA756960	SAMN20930930	423	93769	496466	5088262
C6580	Escherichia coli	PRJNA756960	SAMN20930931	436	95184	496464	5176618
C6592	Escherichia coli	PRJNA756960	SAMN20930932	332	88273	470011	4961137
C6599	Escherichia coli	PRJNA756960	SAMN20930933	366	93320	464624	5156703
C6617	Escherichia coli	PRJNA756960	SAMN20930934	322	88273	363528	5005902
C6619	Escherichia coli	PRJNA756960	SAMN20930935	363	73942	274101	4786618

C6620	Escherichia coli	PRJNA756960	SAMN20930936	293	75856	274101	4772111
